# A newly-recorded species of the genus *Ablemma* Roewer, 1963 (Araneae, Tetrablemmidae) from China

**DOI:** 10.3897/BDJ.10.e85365

**Published:** 2022-05-11

**Authors:** Hongjin Fu, Yuzhu Wang, Yanfeng Tong

**Affiliations:** 1 College of Life Science, Shenyang Normal University, Shenyang 110034, China College of Life Science, Shenyang Normal University Shenyang 110034 China

**Keywords:** Asia, Guangdong, spider, taxonomy, tetrablemmids

## Abstract

**Background:**

*Ablemma* Roewer, 1963 is a species-rich genus of the family Tetrablemmidae O. Pickard-Cambridge, 1873, currently comprising 28 known species. This genus is mainly distributed in Southeast Asia. Currently, only one species, *A.prominens* Tong & Li, 2008 is known to occur in China.

**New information:**

The species *Ablemmashimojanai* (Komatsu, 1968), presently only known from the Ryuku Islands in Japan, is reported from China for the first time on the basis of material collected in Guangdong Province. A morphological description and detailed images are provided.

## Introduction

Tetrablemmidae O. Pickard-Cambridge is a small family of spiders known from tropical and subtropical regions. They are haplogyne, ecribellate spiders diagnosed from most other families by the presence of complex abdominal scuta (Jocqué and Dippenaar-Schoeman 2006). They are small (0.8–2.0 mm), cryptic spiders predominantly living in leaf litter, soil and caves (Burger et al. 2010).

Tetrablemmidae currently includes 150 species in 27 genera worldwide (WSC 2022), of which eight genera and 20 species occur in China: *Ablemma* Roewer, 1963 (1 sp.), *Brignoliella* Shear, 1978 (3 spp.), *Indicoblemma* Bourne, 1980 (1 sp.), *Lehtinenia* Tong & Li, 2008 (2 spp.), *Shearella* Lehtinen, 1981 (1 sp.), *Sinamma* Lin & Li, 2014 (3 spp.), *Singaporemma* Shear, 1978 (4 spp.) and *Tetrablemma* O. Pickard-Cambridge, 1873 (5 spp.) (Tong and Li 2008, Lin and Li 2010, Lin and Li 2014, Ballarin et al. 2021, He and Lin 2021, Cheng et al. 2022).

*Tetrablemmashimojanai* Komatsu, 1968 was described, based on a single male collected in Inamiji Cave in Okinawa, Japan (Komatsu 1968) and was later transferred to the genus *Ablemma* Roewer, 1963 by Shear (1978). This species is widely distributed in the Ryukyu Chain and often can be found in limestone caves (Shimojana 1977). A recent investigation revealed that *A.shimojanai* also occurs in the leaf litter of deciduous forests (Suzuki et al. 2018). However, this species is still poorly known. Details of the male palp are currently known, based on drawings only (Komatsu 1968, Shear 1978) and no images exist of the internal female genitalia.

In this paper, *Ablemmashimojanai* is recorded from China for the first time and a detailed description and illustrations of this species are provided.

## Materials and methods

The specimens used in this study were collected by sifting leaf litter and later examined using a Leica M205C stereomicroscope. Photos were made with a Canon EOS 750D zoom digital camera (18 megapixels) mounted on an Olympus BX51 compound microscope. Scanning electron microscope (SEM) images were taken under high vacuum with a Hitachi TM3030 after critical-point drying and gold-palladium coating. All measurements were taken using an Olympus BX51 compound microscope and are given in millimetres.

The specimens are deposited in Shenyang Normal University (SYNU).

## Taxon treatments

### Ablemma shimojanai

(Komatsu, 1968)

81B9C4A7-0E1B-540A-BBA5-F3CDF763D3EB

#### Materials

- **Type status:** Other material. **Occurrence:** individualID: SYNU-506; individualCount: 2; sex: 1 male, 1 female; lifeStage: adult; **Taxon:** scientificName: *Ablemmashimojanai* (Komatsu, 1968); order: Araneae; family: Tetrablemmidae; genus: Ablemma; **Location:** country: China; stateProvince: Guangdong; county: Shaoguan City; locality: Zhangjiuling Forest Park; verbatimElevation: 200 m; verbatimCoordinates: 24°48'27"N, 113°32'44"E; **Identification:** identifiedBy: Yanfeng Tong; **Event:** samplingProtocol: sifting leaf litter; eventDate: 8 April 2021
- **Type status:** Other material. **Occurrence:** individualID: SYNU-507; individualCount: 5; sex: 3 males, 2 females; lifeStage: adult; **Taxon:** scientificName: *Ablemmashimojanai* (Komatsu, 1968); order: Araneae; family: Tetrablemmidae; genus: Ablemma; **Location:** country: China; stateProvince: Guangdong; county: Jieyang City; locality: Huangqishan Forest Park; verbatimElevation: 120 m; verbatimCoordinates: 23°34'12"N, 116°22'15"E; **Identification:** identifiedBy: Yanfeng Tong; **Event:** samplingProtocol: sifting leaf litter; eventDate: 18 April 2021

#### Description

**Figure 1.**
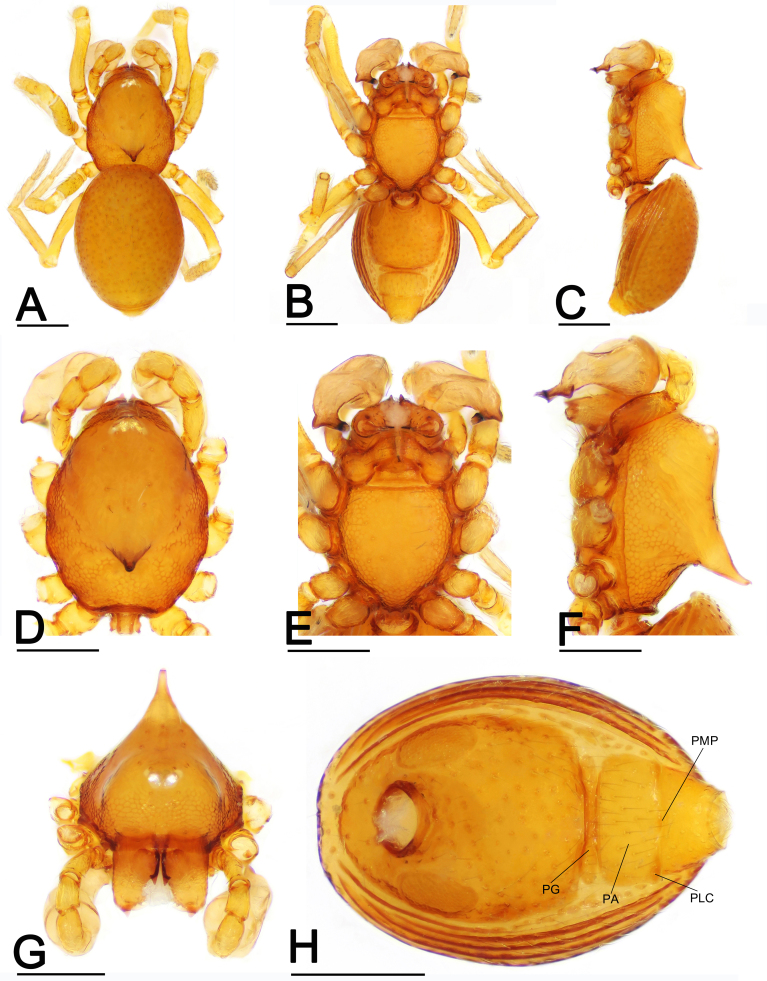
*Ablemmashimojanai* (Komatsu, 1968), male. **A** habitus, dorsal view; **B** habitus, ventral view; **C** habitus, lateral view; **D** prosoma, dorsal view; **E** prosoma, ventral view; **F** prosoma, lateral view; **G** prosoma, anterior view; **H** abdomen, ventral view. Abbreviations: PA = preanal plate; PG = postgenital plate; PLC = posterolateral corners; PMP = posteromedial projection. Scales: 0.2 mm.

**Male.** Habitus as in Fig. 1A-C. Body brownish-yellow. Total length 0.97; carapace 0.51 long, 0.35 wide; abdo­men 0.61 long, 0.43 wide. Prosoma (Fig. 1D and F): carapace finely reticulate; 4 eyes, white, strongly recurved as seen from above; clypeus high, sloping for­ward, marginally rounded; cephalic part raised, posterior part with large conical projection; chelicerae robust, with small basal projection on anterior surface of paturon and an anterodistal tooth (Fig. 1G), cheliceral lamina well developed; labium triangular, blunt distally; sternum finely reticulated, with sparse setae (Fig. 1E). Legs yellowish-orange.

Opisthosoma (Fig. 1A-C and H): dorsal scutum oval, dimpled with tiny pits, smooth between pits, covered with sparse setae; ventral scutum rugose; perigenital plate absent; postgenital plate (PG) straight, nearly same width as preanal plate (PA), 1/4 of the preanal plate length; preanal plate rectangular, with thick posterolateral corners (PLC) and posteromedial projection (PMP).

Palp (Fig. 3A-I): femur (FE) approxi­mately 2 times longer than patella (PAT); tibia (TI) not swollen, with dorsal trichobo­thrium distally; cymbium (CY) small, cup-shaped; bulb (BU) long pear-shaped, surface smooth; sper­m duct (SD) broad basally, narrow distally; embolus (EM) short, foot-shaped, strongly sclerotised distally, with small acute tip.

**Figure 2.**
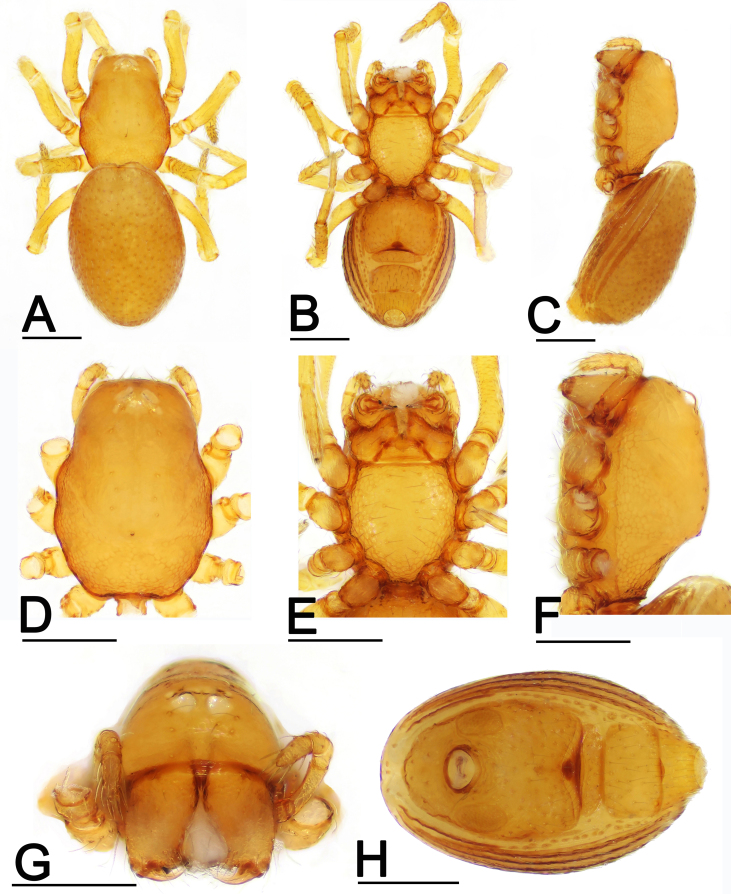
*Ablemmashimojanai* (Komatsu, 1968), female. **A** habitus, dorsal view; **B** habitus, ventral view; **C** habitus, lateral view; **D** prosoma, dorsal view; **E** prosoma, ventral view; **F** prosoma, lateral view; **G** prosoma, anterior view; **H** abdomen, ventral view. Scales: 0.2 mm.

**Female**. Habitus as in Fig. 2A-C. Total length 0.96; carapace 0.44 long, 0.32 wide; abdomen 0.65 long, 0.41 wide. Cephalic part lacking large conical projection, chelicerae unmodified; epigynal fold (EF) distinct; other features as in male.

**Figure 3.**
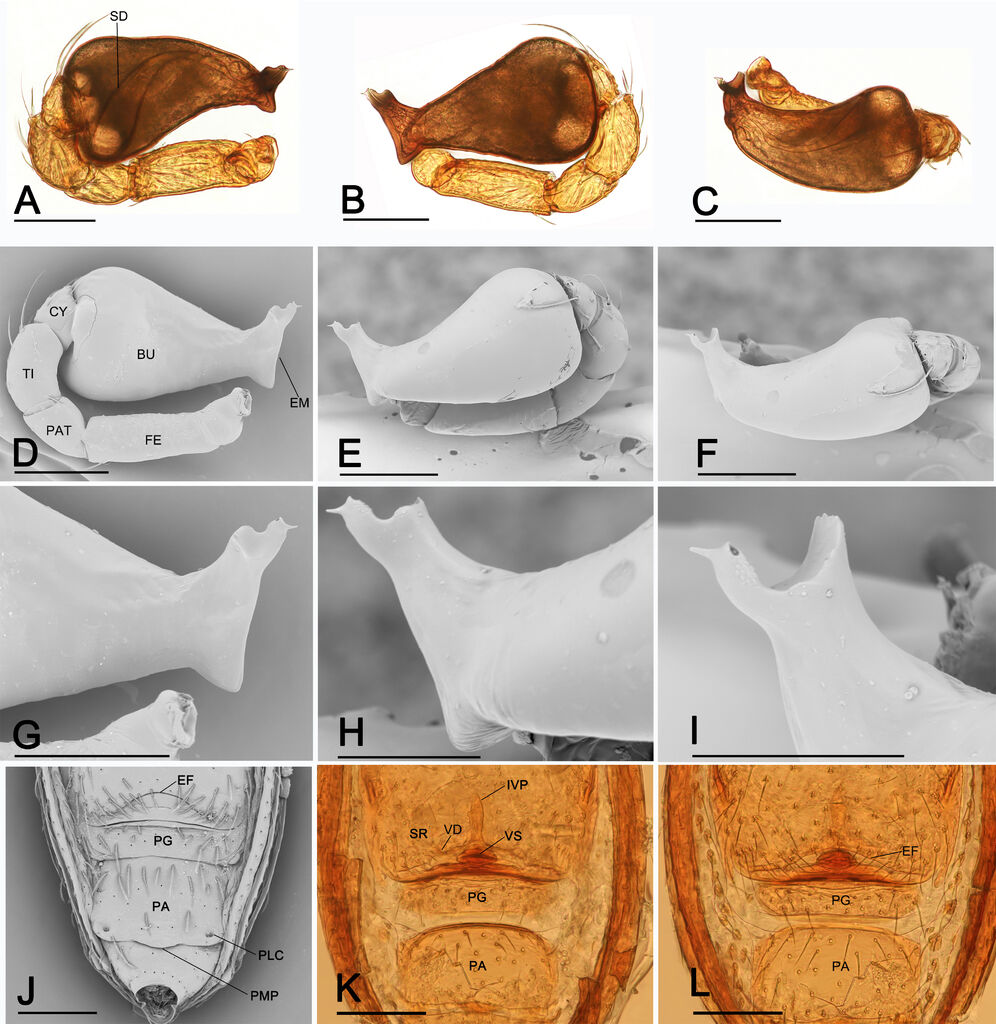
*Ablemmashimojanai* (Komatsu, 1968), male, A-C (light) and D-I (SEM); female, J (SEM) and K, L (light). **A** left palp, prolateral view; **B** left palp, retrolateral view; **C** left palp, dorsal view; **D** left palp, prolateral view; **E** left palp, retrolateral view; **F** left palp, dorsal view; **G** distal part of bulb, prolateral view; **H** distal part of bulb, retrolateral view; **I** distal part of bulb, dorsal view; **J** genital area, ventral view; **K** genital area, dorsal view; **L** genital area, ventral view. Abbreviations: BU = bulb; CY = cymbium; EF = epigynal fold; EM = embolus; FE = femur; IVP = inner vulval plate; PA = preanal plate; PAT = patella; PG = postgenital plate; PLC = posterolateral corners; PMP = posteromedial projection; SD = sperm duct; SR = seminal receptaculum; TI = tibia; VD = vulval duct; VS = vulval stem. Scales: A-G, J-L = 0.1 mm; H, I = 0.05 mm.

Genitalia (Fig. 3J-L): vulval stem (VS) forming an oval structure, strongly sclerotised; vulval duct (VD) weakly sclerotised, con­nected to translucent, saccular seminal receptaculum (SR); inner vulval plate (IVP) long; central process absent.

#### Diagnosis

This species is similar to *Ablemmaberryi* Shear, 1978 in the large conical projection of male carapace (cf. Fig. 1C and F and Shear 1978: fig. 90), but can be distinguished by the acute tip of the embolus (Fig. 3A-I; vs. very long, laminar embolus, see Shear 1978: figs 93 and 94) and the large preanal plate, which is nearly four times the length of the postgenital plate (Fig. 1H and Fig. 2H; vs. two times the length of the postgenital plate, see Shear 1978: fig. 95).

#### Distribution

China (Guangdong), Japan (Ryukyu Island).

## Supplementary Material

XML Treatment for Ablemma shimojanai

## References

[B7825875] Ballarin F., Yamasaki T., Su Y. (2021). A survey on poorly known rainforest litter-dwelling spiders of Orchid Island (Lanyu, Taiwan) with the description of a new species (Araneae: Linyphiidae, Tetrablemmidae, and Theridiosomatidae). Zootaxa.

[B7825884] Burger M., Harvey M., Stevens N. (2010). A new species of blind subterranean *Tetrablemma* (Araneae: Tetrablemmidae) from Australia. Journal of Arachnology.

[B7825893] Cheng W., Ren L., Tong Y., Bian D., Li S. (2022). Two new species of the spider genus *Sinamma* Lin & Li, 2014 (Araneae, Tetrablemmidae) from Guangdong Province, China. Zootaxa.

[B7825903] He Y., Lin Y. (2021). Two new armored spiders (Araneae, Tetrablemmidae) from Yunnan, China. Acta Arachnologica Sinica.

[B7825912] Jocqué R., Dippenaar-Schoeman A. (2006). Spider families of the world. Musée Royal de l'Afrique Central,.

[B7825921] Komatsu T. (1968). Two new cave spiders of genera *Tetrablemma* (Tetrablemminae, Oonopidae) and *Dolichocybaeus* (Cybaeinae). Acta Arachnologica.

[B7825930] Lin Y., Li S. (2010). New armored spiders of the family Tetrablemmidae from China. Zootaxa.

[B7825939] Lin Y., Li S. (2014). New cave-dwelling armored spiders (Araneae, Tetrablemmidae) from Southwest China. ZooKeys.

[B7825948] Shear W. (1978). Taxonomic notes on the armored spiders of the families Tetrablemmidae and Pacullidae. American Museum Novitates.

[B7825957] Shimojana M. (1977). Preliminary report on the cave spider fauna of the Ryukyu Archipelago. Acta Arachnologica.

[B7825983] Suzuki Y., Iida K., Sawahata T., Hayasaka D. (2018). Spiders collected with pitfalls trap at Kuchinoerabu Island. Kishidaia.

[B7825966] Tong Y., Li S. (2008). Tetrablemmidae (Arachnida, Araneae), a spider family newly recorded from China. Organisms Diversity & Evolution.

[B7825975] WSC World Spider Catalog. Version 23.0. Natural History Museum Bern. http://wsc.nmbe.ch.

